# Right Ventricular Dysfunction following Acute Myocardial Infarction in the Absence of Pulmonary Hypertension in the Mouse

**DOI:** 10.1371/journal.pone.0018102

**Published:** 2011-03-24

**Authors:** Stefano Toldo, Herman J. Bogaard, Benjamin W. Van Tassell, Eleonora Mezzaroma, Ignacio M. Seropian, Roshanak Robati, Fadi N. Salloum, Norbert F. Voelkel, Antonio Abbate

**Affiliations:** VCU Pauley Heart Center and Victoria Johnson Research Center, Virginia Commonwealth University, Richmond, Virginia, United States of America; University of Modena and Reggio Emilia, Italy

## Abstract

**Background:**

Cardiac remodelling after AMI is characterized by molecular and cellular mechanisms involving both the ischemic and non-ischemic myocardium. The extent of right ventricular (RV) dilatation and dysfunction and its relation to pulmonary hypertension (PH) following AMI are unknown. The aim of the current study was to evaluate changes in dimensions and function of the RV following acute myocardial infarction (AMI) involving the left ventricle (LV).

**Methods:**

We assessed changes in RV dimensions and function 1 week following experimental AMI involving the LV free wall in 10 mice and assessed for LV and RV dimensions and function and for the presence and degree of PH.

**Results:**

RV fractional area change and tricuspidal annular plane systolic excursion significantly declined by 33% (P = 0.021) and 28% (P = 0.001) respectively. Right ventricular systolic pressure measured invasively in the mouse was within the normal values and unchanged following AMI.

**Conclusion:**

AMI involving the LV and sparing the RV induces a significant acute decline in RV systolic function in the absence of pulmonary hypertension in the mouse indicating that RV dysfunction developed independent of changes in RV afterload.

## Introduction

Acute myocardial infarction (AMI) is associated with compensatory mechanisms involving both the left and right ventricles. In experimental models of AMI, right ventricular (RV) hypertrophy occurs even if the RV is initially spared [1]. Hypertrophy and apoptosis of cardiomyocytes both at the site of AMI and at remote unaffected sites of both the LV and the RV are seen in biventricular remodeling following AMI [1]–[5]. In patients with LV systolic dysfunction and heart failure the presence of signs and/or symptoms of RV failure identify a subgroup of patients with a very poor prognosis [6]–[10]. The mechanisms leading to RV remodelling and dysfunction following AMI involving the LV are not completely clear, but it is frequently assumed that LV failure causes pulmonary hypertension (PH) and increased RV afterload leading to RV remodelling and dysfunction. In addition, infarction or ischemia of the RV and/or the septum are common in patients with AMI and can also contribute to abnormal RV systolic function. However studies concerning RV remodelling are few and the extent, time and causes for RV dilatation and dysfunction remain unclear [11]. In the current study we evaluated the occurrence and extent of RV dilatation and dysfunction in a mouse model of AMI in which the septum and RV are spared thus eliminating the possibility that RV or septal ischemia/infarction leads to abnormal RV perfusion or mechanics, and in which the RV systolic pressure could be invasively measured. We hypothesized that increased RV afterload due to PH leads to impaired RV function following AMI.

## Methods

### Experimental AMI

**Figure 1:**
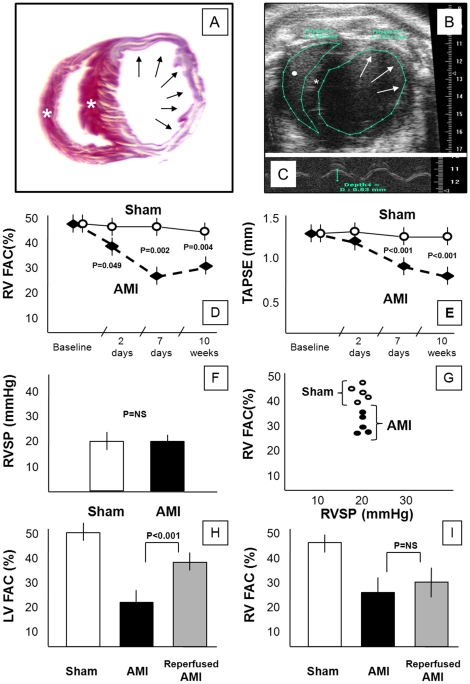
Right ventricular remodeling in AMI. Panel A shows cross-section of the left and right ventricles (apical section) stained with Masson's trichrome to identify fibrous scar in the infarct. Involvement of a large area of the anterolateral left ventricle free wall (arrows) and sparing of the interventricular septum and right ventricle (*) is evident. Panel B shows an echocardiographic image (short axis view) of the left and right ventricles (mid-ventricular section) in a mouse 7 days after permanent coronary artery ligation. An aneurysm of the anterolateral left ventricular free wall is noted (arrows). The interventricular septum is indicated by (*) and the right ventricular cavity is indicated by (•). Panel C shows a M-mode recording on the tricuspidal annulus plane systolic excursion (TAPSE) obtained from a 4-chamber apical view. Panels D and E show changes in right ventricular fractional area change (RVFAC) and tricuspidal annulus plane systolic excursion (TAPSE) over time in mice with AMI due to permanent coronary ligation and mice with sham operation (N = 10 per group). Panel F shows right ventricular systolic pressure (RVSP) in mice with AMI due to permanent coronary ligation and mice with sham operation 7 days after surgery with no differences noted between the groups (N = 5 per group). Panel G shows the lack of correlation between RVSP and RVFAC in mice with AMI due to permanent coronary ligation and mice with sham operation 7 days after surgery. Panels H and I show data deriving from the model of reperfused AMI as it compares with the non-reperfused AMI: reperfused AMI had a smaller decline in LV systolic function (LVFAC, panel H) yet a similar decline in RV systolic function (RVFAC, panel I).

Adult male out-bred ICR mice were purchased from Harlan Laboratories (Indianapolis, IN). All animal experiments were conducted under the guidelines on humane use and care of laboratory animals for biomedical research published by the US National Institutes of Health (NIH Publication No. 85–23, revised 1996). The study protocol was approved by the local Institutional Animal Care and Use Committee. Mice underwent experimental myocardial infarction due to permanent coronary artery ligation or sham surgery as previously described [12]. All mice underwent transthoracic echocardiography before surgery and at 2 and 7 days and a subgroup of mice also 10 weeks after surgery. Doppler echocardiography was performed with the Vevo770 imaging system (VisualSonics Inc, Toronto, Canada) as previously described and measurements were performed according to the to the American Society of Echocardiography recommendations [13]. The left ventricular (LV) end-diastolic diameter (LVEDD), LV end-systolic diameters (LVESD) were measured at M-mode. LV fractional shortening (LVFS) was calculated. The LV and right ventricle (RV) diastolic and systolic areas were traced in the parasternal short axis mid-ventricular view (Figure 1). The LV and RV fractional area change velocity (LVFAC and RVFAC, respectively), a load- and rate-independent indexes of contractility, were computed dividing the fractional area changes by the rate-corrected ejection time. The tricuspidal annular plane systolic excursion was also measured to quantify right ventricular function. To evaluate for interventricular dyssynchrony, we measured the electromechanical delay using (*a*) Doppler analysis of the pulmonary artery (for the RV) and of the aorta (for the LV) measuring the time between the start of the QRS on the electrocardiogram and the start of the forward flow, and (*b*) using the M-mode echocardiography of the mitral annulus plane and tricuspidal annulus plane systolic excursion and measuring the time between the start of the QRS on the electrocardiogram and the start of the inward motion of the lateral portion of the annulus. The differences between the LV and RV delays were used as measures of dyssynchrony. The investigator performing and reading the echocardiogram was blinded to the group allocation.

In an additional group of mice (ligation or sham [N = 5] per group) were deeply sedated and intubated 7 days after surgery, the chest was reopened, the RV or LV apex were punctured and a Millar catheter connected to a pressure transducer to measure RV peak systolic pressure, which is equivalent to pulmonary artery systolic pressure in the absence of pulmonary valve stenosis, or LV end-diastolic pressure. An additional group of mice (ligation or sham [N = 5] per group) underwent measurement of RV peak systolic pressure or LV end-systolic pressure after challenge with 0.4 ml of NaCl 0.9% or isoproterenol 10 ng (Sigma Aldrich, St. Louis, MO, US) to evaluate for latent PH.

The extent of the infarct was evaluated using two different methods. Triphenyl tetrazolium chloride (TTC) was used to stain viable myocardium samples at 24 hours and Masson's trichrome histochemical staining for the measurement of infarct size as reparative fibrosis (scar), as previously described [14] (Figure 1).

The presence of pulmonary edema was assessed by measuring lung water content 7 days after AMI: the lungs were dissected, weighed, dried at 60°C (24 hrs), and then re-weighed to calculate wet-to-dry ratio.

As an additional AMI model, we added a group of mice (N = 6), which underwent transient coronary artery ligation (30 min) followed by reperfusion in order to simulate a reperfused AMI, often seen in patients, which is also characterized by a smaller degree of LV injury. The surgical technique is as described above with the difference that the ligation was released 30 min later. These mice underwent echocardiography prior to surgery and 7 days later.

### Statistical analysis

The values are reported as mean and standard error. ANOVA for repeated measure analysing the effect of time, group and time x group interaction was used to compare variables between the animal groups. The analyses were completed using SPSS 16.0 software (Chicago, IL).

## Results

### Experimental AMI

Surgical ligation of the left coronary artery was associated with a significant reduction in LVFS, LVEF and LV fractional area change at 48 hours, 7 days and 10 weeks after surgery and a significant increase in LV dimensions (LVEDD, LVESD, LV diastolic area and LV systolic area) by day 7. At day 7, LV fractional area change velocity (a load- and rate-independent index of contractility) decreased by 45% (P<0.001). Cardiac output estimated by Doppler did not significantly change reflecting a condition of compensated LV dysfunction (Table 1).

**Table 1:** Experimental AMI in the mouse.

	SHAM		AMI			
	Baseline	48 h	7 days	Baseline	48 h	7days
LVFS (%)	36	34	35	35	21*	15*
LVEF (%)	68	64	67	66	43*	31*
LVEDD (mm)aa	3.65	3.68	3.72	3.74	3.78	4.75*
LVESD (mm)	2.27	2.36	2.28	2.42	2.98*	4.15*
LV diastolic area (mm^2^)	10.4	10.1	10.6	10.6	15.0*	17.4*
LV systolic area (mm^2^)	5.1	5.7	5.4	5.2	10.1*	13.5*
LVFAC (%)	52	48	49	52	33*	23*
LVFAC velocity (%/ms)	0.30	0.29	0.31	0.31	0.21*	0.16*
Cardiac output (ml/min)	13.8	14.1	12.7	15.2	13.8	12.7
LVEDP (mmHg)	-	-	3	-	-	4
RV diastolic area (mm^2^)	4.6	4.7	4.5	4.7	4.8	5.7*
RV systolic area (mm^2^)	2.4	2.7	2.5	2.4	3.1	4.0*
RVFAC (%)	47	45	47	48	37	28*
RVFAC velocity (%/sec)	0.27	0.25	0.25	0.26	0.22*	0.19*
TAPSE (mm)	1.25	1.15	1.40	1.24	1.08	0.90*
RVSP (mmHg)	-	-	20	-	-	20

Footnote for Table 1.

*P<0.05 vs baseline.

**Abbreviations:** LV- Left ventricular; LVEDD – Left Ventricular End-diastolic Diameter; LVESD – Left Ventricular End-systolic Diameter; LVEF – Left Ventricular Ejection Fraction; LVFAC – Left Ventricular Fractional Area Change; LVFS – Left ventricular fractional shortening; LVEDP – Left ventricular end diastolic pressure; RV- Right ventricular; RVFAC – Right Ventricular Fractional Area Change; RVSP – Right ventricular systolic pressure; TAPSE – Tricuspidal Annulus Plane Systolic Excursion.

RV fractional area change was significantly reduced at 48 hours (−21%, P = 0.049), 7 days (−39%, P = 0.002), and 10 weeks (−28%, P = 0.004), while RV diastolic area significantly increased by day 7 (+20%, P<0.001)(Figure 1, Table 1). At day 7, RV fractional area change velocity decreased by 28% (P = 0.042)(Figure 1, Table 1). TAPSE was also significantly reduced at day 7 (−27%, P<0.001, Figure 1, Table 1), however the changes in tricuspidal annulus systolic velocity at tissue Doppler did not reach statistical significance.

There was no evidence of interventricular dyssynchrony 7 days after surgery: the Doppler measured delay difference between aorta (LV) and pulmonary artery (RV) was 2.6±0.3 ms in the AMI group and 2.3±0.4 ms in the sham-operated mice (P = 0.67), the M-Mode measured delay difference between the mitral annulus plane (LV) and the tricuspidal annulus plan (RV) was 0±0.6 in the AMI group and 1.6±1.0 ms in the sham-operated mice (P = 0.27).

Invasively measured RV systolic pressure and LV end-diastolic pressure were virtually unchanged after AMI (20±1 mmHg [sham] vs 20±2 [AMI], P = 0.92; and 3±1 mmHg [sham] vs 4±1 [AMI], P = 0.76; respectively), and unchanged after NaCl 0.9% or isoproterenol challenge (data not shown). The lung water content was not increased after AMI reflecting absence of pulmonary edema (78±0.8% vs 77.8±0.8% in the sham operated mouse, P = 0.22).

The extent of infarct size was similar using both TTC (29±3%) and Masson's trichrome (28%±3%), and in all cases the infarct involved only the anterior and lateral wall while the RV and septum were not involved.

Mice with reperfused AMI showed a significantly smaller infarct size using both TTC (14±2%, P<0.001 vs non-reperfused) and Masson's trichrome (13±3%, P<0.001 vs non-reperfused) and a smaller decrease in LVFAC (38±4%, P<0.001 vs non-reperfused) yet a similar reduction in RVFAC (30±5%, P = 0.48, Figure 1).

The changes in RVFAC were not statistically correlated with the size of infarct or the changes in LVFAC in the non-reperfused AMI group, in the reperfused AMI group, or in the 2 groups combined (data not shown), whereas LVFAC correlated significantly with infarct size in the 2 groups combined (R = −0.86, P<0.01) but not in the individual groups. RVFAC and LVFAC as absolute values were highly correlated in the 2 groups combined (R = +0.97, P<0.01) but not in the individual groups.

## Discussion

Post-infarction cardiac remodelling is characterized by biventricular remodelling. The occurrence of RV dysfunction and failure identifies a subgroup of patients with extremely poor prognosis, however only few clinical studies have systematically addressed RV remodelling following AMI [7]–[9], [15]. Potential mechanisms include ischemia/infarct of the RV, septal dyssynergy, pulmonary hypertension, neurohormonal activation or inflammation [11], [16]. Our data shows that a significant degree of RV systolic dysfunction ensues acutely after AMI in the absence of RV ischemia/infarct or septal infarct, and in the absence of pulmonary hypertension. These data are in accordance to data obtained in patients with chronic heart failure in which progressive RV enlargement occurred in the absence of PH [16]–[17], and call for a refocusing on cellular and molecular events occurring in the RV myocardium rather than in the pulmonary circulation when trying to explain the development of RV failure [11], [17]. These data seem also to imply that the LV and RV may be working as a ‘functional syncytium’ and that the two ventricles cannot be dissociated in an independent manner since the architecture of the distinct myocardial bands makes it mandatory for an integrated and unified function of both chambers [18]–[19]. Seminal work in the isolated rabbit heart (independently of pulmonary circulation and neural, humoral and pericardial influences) had showed that reduced LV systolic function induced by a damage to the LV free wall inevitably led to RV systolic dysfunction [19], we confirm and expand such findings by showing that RV systolic dysfunction occurs in vivo in the mouse independent of changes in RV afterload. We also report that the degree in RV dysfunction is not linearly correlated with LV infarct size. The reasons why neither LV end-diastolic pressure nor RV systolic pressure increase after AMI in the mouse are not clear, yet serve well as a model to understand RV remodelling in the absence of PH.

RV remodelling and dysfunction following AMI may therefore simply be part of a global biventricular remodelling response due to altered wall stress which is further aggravated by neurohormonal activation during and after AMI [16]–[17], [20]. LV and RV failure may be independently or concomitantly contributing to heart failure, as reflected by the fact that LV systolic function and RV systolic function are closely correlated. RV trabeculae from rats with AMI involving the LV showed impaired calcium handling and contractility independent of ischemia and prevented by angiotensin receptor blockade [20]. Similarly, improvements in LV and RV systolic function occur with carvedilol in patients with chronic systolic heart failure [16]. In the SAVE and VALIANT studies enrolling patients with recent AMI and LV systolic dysfunction, each 5% decrease in RV fractional area change (a surrogate for RV ejection fraction) was associated with a 16–30% increased odds of cardiovascular mortality [6], [10].

In conclusion, acute myocardial infarction involving the LV and sparing the septum and the RV induces a significant acute decline in RV systolic function in the absence of pulmonary hypertension in the mouse. RV systolic dysfunction following AMI may represent an additional mechanism by which AMI may cause heart failure and increased mortality. These finding remain to be validated and to be mechanistically investigated, ultimately leading to clinical studies addressed at determining whether RV systolic function is an independent and modifiable predictor of mortality.

## References

[1] Patten RD, Aronovitz MJ, Deras-Meja L, Pandian NG, Hanak GG (1998). Ventricular remodeling in a mouse model of myocardial infarction.. Am J Physiol.

[2] Abbate A, Bussani R, Sinagra G, Barresi E, Pivetta A (2008). Right ventricular cardiomyocyte apoptosis in patients with acute myocardial infarction of the left ventricular wall.. Am J Cardiol.

[3] Anversa P, Olivetti G, Capasso JM (1991). Cellular basis of ventricular remodeling after myocardial infarction.. Am J Cardiol.

[4] Bussani R, Abbate A, Biondi-Zoccai GG, Dobrina A, Leone AM (2003). Right ventricular dilatation after left ventricular acute myocardial infarction is predictive of extremely high peri-infarctual apoptosis at postmortem examination in humans.. J Clin Pathol.

[5] Pfeffer MA, Braunwald E (1990). Ventricular remodeling after myocardial infarction – Experimental observations and clinical implications.. Circulation.

[6] Anavekar NS, Skali H, Bourgoun M, Ghali JK, Kober L (2008). Usefulness of right ventricular fractional area change to predict death, heart failure and stroke following myocardial infarction (from the VALIANT ECHO study).. Am J Cardiol.

[7] Di Salvo TG, Mathier M, Semigran MJ, Dec GW (1995). Preserved right ventricular ejection fraction predicts exercise capacity and survival in advanced heart failure.. J Am Coll Cardiol.

[8] Larose E, Ganz P, Reynolds G, Dorbala S, Di Carli MF (2007). Right ventricular dysfunction assessed by cardiovascular magnetic resonance imaging predicts poor prognosis late after myocardial infarction.. J Am Coll Cardiol.

[9] Oakley C (1998). Importance of right ventricular function in congestive heart failure.. Am J Cardiol.

[10] Zornoff LAM, Skali H, Pfeffer MA, Sutton MSJ, Rouleau JL (2002). Right ventricular dysfunction and risk of heart failure and mortality after myocardial infarction.. J Am Coll Cardiol.

[11] Haddad F, Hunt SA, Rosenthal DN, Murphy DJ (2008). Right ventricular function in cardiovascular disease, part I.. Circulation.

[12] Abbate A, Salloum F, Vecile E, Das A, Hoke N, Straino S (2008). Anakinra, a recombinant human interleukin-1 receptor antagonist, inhibits apoptosis in experimental acute myocardial infarction.. Circulation.

[13] Gardin J, Adams D, Douglas P, Feigenbaum H, Forst D (2002). Recommendations for a standardized report for adult transthoracic echocardiography: a report from the American Society of Echocardiography's Nomenclature and Standards Committee and Task Force for a Standardized Echocardiography Report.. J Am Soc Echocardiography.

[14] Seropian IM, Abbate A, Toldo S, Harrington J, Smithson L (2010). Pharmacological Inhibition of Phosphoinositide 3-Kinase Gamma (PI3Kγ) Promotes Infarct Resorption and Prevents Adverse Cardiac Remodeling after Myocardial Infarction in Mice.. J Cardiovasc Pharmacol.

[15] Antoni  ML, Scherptong RHC, Atary JZ, Boersma E, Holman ER Circ Cardiovasc Imaging 2010.

[16] Quaife RA, Christian PE, Gilbert EM, Datz FL, Volkman K (1998). Effects of carvedilol on right ventricular function in chronic heart failure.. Am J Cardiol.

[17] Voelkel NF, Quaife RA, Leinwand LA, Barst RJ, McGoon MD (2006). Right ventricular function and failure: report of a National Heart, Lung, and Blood Institute working group on cellular and molecular mechanisms of right heart failure.. Circulation.

[18] Bakos ACP (1950). The question of the function of the right ventricular myocardium: an experimental study.. Circulation.

[19] Santamore WP, Lynch PR, Heckman JL, Bove AA, Meier GD (1976). Left ventricular effects on right ventricular developed pressure.. J Appl Physiol.

[20] Daniels MCG, Keller RS, De Tombe PP (2001). Losartan prevents contractile dysfunction in rat myocardium after left ventricular myocardial infarction.. Am J Physiol.

